# Weight Gain and Liver Steatosis in Patients with Inflammatory Bowel Diseases

**DOI:** 10.3390/nu11020303

**Published:** 2019-02-01

**Authors:** Rocco Spagnuolo, Tiziana Montalcini, Daniele De Bonis, Yvelise Ferro, Cristina Cosco, Elisa Mazza, Stefano Romeo, Patrizia Doldo, Arturo Pujia

**Affiliations:** 1 Department of Clinical and Experimental Medicine, University Magna Grecia, 88100 Catanzaro, Italy; spagnuolo@unicz.it (R.S.); daniele.db@gmail.com (D.D.B.); cosco.cristina@libero.it (C.C.); doldo@unicz.it (P.D.); 2 Department of Medical and Surgical Science, Nutrition Unit, University Magna Grecia, 88100 Catanzaro, Italy; yferro@unicz.it (Y.F.); elisamazza@inwind.it (E.M.); romeo@unicz.it (S.R.); pujia@unicz.it (A.P.); 3 Department of Molecular and Clinical Medicine, University of Gothenburg, 40530 Gothenburg, Sweden

**Keywords:** inflammatory bowel diseases, weight gain, hepatic steatosis, transient elastography

## Abstract

Background and Aim: Most studies focused on the benefits of weight loss on hepatic steatosis and no studies have been specifically designed to assess the role of weight gain on the development of liver steatosis in patients affected by inflammatory bowel diseases. The aim of this study was to analyse the relation between weight change over time and liver steatosis in patients with inflammatory bowel diseases. Methods: We retrospectively evaluated a population of 89 ambulatory patients in clinical remission or affected by mild disease, as determined from disease activity indices, with at least one follow-up visit. Transient elastography was used to quantify liver steatosis. Results: A total of 49 individuals (55%) were overweight/obese at baseline. A significant difference in weight change was found between participants that improved, were stable and worsened, over a mean follow-up of four years. (−1.0 kg ± 4; 2.5 kg ± 6; and 5.4 kg ± 5; respectively, *p* = 0.009). We found a greater probability of worsening in the hepatic fat content in individuals who gained more than 6% of body weight than in those gaining less than this value (log–rank (Mantel–Cox) χ^2^ test = 9.85; df = 1; *p* = 0.002). Conclusions: A body weight gain of 6% increases the probability of deterioration in liver steatosis over a period of four years in patients with inflammatory bowel diseases. Weight gain prevention with lifestyle interventions may be the cornerstone treatment of these patients.

## 1. Introduction 

Liver steatosis is linked to obesity, with a prevalence rate of 80% in extremely obese patients [1,2]. The cellular mechanisms responsible for the accumulation of intrahepatic triglyceride (IHTG) over time is complex and not completely clear [3]. However, the most important pathological mechanism in hepatic steatosis involves the secretion of proinflammatory molecules (such as cytokines and adipokines) causing not only several systemic complications, such as insulin resistance and dyslipidaemia, but also liver disease progression. In obese individuals both increased lipolysis in visceral adipose tissue and a high-fat diet can induce chronic intracellular inflammation in the liver by the inhibition of the nuclear factor kappa-B kinase subunit beta/nuclear factor kappa-B (IKK-β/NF-κB) signalling pathway [4,5,6]. Interestingly, it has been demonstrated that the suppression of the inflammation can ameliorate hepatic steatosis [7].

All these pathophysiological mechanisms have provided the basis for understanding the findings of several epidemiological studies regarding the association between inflammatory bowel disease (IBD) and an increased risk for the development of non-alcoholic fatty liver disease (NAFLD) [8,9]. 

A recent study reports a NAFLD prevalence of 33% in IBD patients, assessed by transient elastography (TE) [10]. Although an association between liver steatosis and high BMI was reported in these patients [10], competing etiologies, including concomitant medication and comorbidities, currently prevent a single cause from being identified for the development of liver disease in IBD. 

Moreover, no studies have been specifically designed to assess the role of weight gain over time in the development and progression of the liver steatosis in IBD patients. 

The aim of this study was thus to analyse the evolution of liver fat content in relation to the weight change over time in IBD patients. 

## 2. Methods 

In this retrospective single centre study, the population was composed of adult ambulatory IBD patients diagnosed by conventional endoscopic, radiological, and histological criteria, regularly attending the IBD service of the “Mater Domini” Azienda University Hospital in Catanzaro (Italy), between January 2014 and October 2018 with at least one follow-up visit. To better understand each contributing factor, including weight gain and exposure to several drugs, and taking into account that a prolonged period of a metabolically unhealthy status could result in liver damage, in this study patients with IBD and NAFLD were followed up for testing at 4 years from the basal measurement.

A convenience sample of 104 individuals was included in this study. All the participants were in clinical remission or affected by mild disease, as determined by clinical disease activity indices. 

We excluded those who had clinical and laboratory signs of chronic hepatitis B and/or C virus infection, past and current alcohol abuse (>20 g of alcohol per day; 350 mL (12 oz) of beer, 120 mL (4 oz) of wine, and 45 mL (1.5 oz) of hard liquor each contain 10 g of alcohol), impaired liver function, presence of autoimmune or cholestatic liver disease, liver cirrhosis, hypothyroidism, pregnancy, nephrotic syndrome, chronic renal failure, cancer and who had incomplete data as ascertained from their clinical records [11]. 

Basal and follow-up clinical characteristics of the population such as body weight, height and disease activity indices were obtained from clinical records. Furthermore, we obtained data on the liver fat content assessment, biochemical parameters such as glucose, total cholesterol, high density lipoprotein (HDL)-cholesterol, triglycerides, liver enzymes, total bilirubin, C-reactive protein (CRP), insulin and medications at basal from clinical records. 

A sample of 89 individuals with a basal TE assessment was enrolled. A successive instrumental liver assessment was performed on the date when the study was conducted. 

Local ethical committee at the “Mater Domini” Azienda University Hospital approved the protocol (49/2014). Written informed consent was obtained from all participants. The investigation conforms to the principles outlined in the Declaration of Helsinki.

### 2.1. Anthropometric and Cardiovascular Risk Factors Assessment 

We assessed the presence of obesity, diabetes and smoking from clinical records. Body weight was measured before breakfast with a calibrated scale and height measured with a wall-mounted stadiometer. The BMI was calculated with the following equation: weight (kg)/height (m) [2,12]. Obesity was defined by the presence of a body mass index (BMI) ≥ 30 kg/m^2^. We assessed the prevalence of the current smokers defined as who has smoked more than 100 cigarettes in their lifetime and smokes cigarettes every day or some days [13]. The following criteria were used to define diabetes: fasting blood glucose ≥ 126 mg/dL or antidiabetic treatment [11,14].

### 2.2. IBD Activity Indexes

In order to evaluate disease activity at a given time, the Mayo Full Score (MS), the Harvey–Bradshaw Index (HBI), the Simple Endoscopic Score for Crohn’s disease (SES-CD), and Rutgeerts score were assessed. 

The MS is a composite clinical, endoscopic, quality of life and global assessment index of ulcerative colitis activity, widely used in clinical trials. Scores range from 0 to 12 points (no activity, to most severe) [15,16].

The HBI is a numeric index of Crohn’s disease activity requiring a limited physical examination for abdominal mass and patient recall from the previous day. It is composed of 5 parameters: general well-being, abdominal pain, number of liquid stools per day, abdominal mass, and complications. Patients who scored 3 or less are very likely to be in remission while patients with a score of 8 to 9 or higher are considered to have severe disease [17].

The SES-CD is a numeric index of endoscopic disease activity. Active endoscopic disease was defined as SES-CD ≥ 3. Specifically, an SES-CD score of 0–3 was defined as inactive disease, while patients with a score of 17 or higher are considered to have severe disease [18].

The Rutgeerts score is used as the standard evaluation of post-surgical endoscopic recurrences at ileocolic anastomosis level [19].

### 2.3. Liver Transient Elastography 

All enrolled IBD patients obtained liver fat content measurement at basal and follow-up visit at the Clinical Nutrition Unit of the “Mater Domini” Azienda University Hospital in Catanzaro. Transient elastography can quantify liver steatosis by controlled attenuation parameter (CAP) assessment and measure liver stiffness (Fibroscan®; Echosense SASU, Paris, France) [11]. Both stiffness and CAP score were obtained simultaneously and in the same volume of liver parenchyma. All patients were evaluated using the 3.5 MHz standard M probe on the right lobe of the liver through intercostal spaces with the patient lying supine and placing the right arm behind the head to facilitate access to the right upper quadrant of the abdomen. The tip of the probe transducer was placed on the skin between the rib bones at the level of the right lobe of the liver. All scans were performed by the same investigator. Liver stiffness was expressed by the median value (in kPa) of ten measurements performed between 25 and 65 mm depth. Only results with 10 valid shots and interquartile range (IQR)/median liver stiffness ratio < 30% were included. The cut-off value for defining the presence of fibrosis was liver stiffness > 7 kPa. 

We assessed CAP score using only the M probe because the CAP algorithm is specific to this device. Ten successful measurements were performed on each patient, and only cases with ten successful acquisitions were taken into account for this study. The success rate was calculated as the number of successful measurements divided by the total number of measurements. The ratio of the IQR of liver stiffness to the median (IQR/dB/m) was calculated as an indicator of variability. The final CAP score (ranged from 100 to 400 decibels per meter (dBm−), was the median of individual measurements. The ratio of IQR in CAP values to the median (IQR/ M CAP) was used as an indicator of variability for the final CAP. For each patient, examinations were performed by two operators who were blinded to clinical and histological data and who were the same at basal and follow-up examination. Operators were blinded to liver measurement performed by his colleague.

The diagnosis of hepatic steatosis was based on a CAP > 216 dB/m. In order to identify each steatosis grade, three different cut-offs were used: CAP between 216 and 252 dB/m for the diagnosis of S1 grade, CAP between 253 and 296 dB/m for the diagnosis of S2 grade, and CAP > 296 dB/m for the diagnosis of S3 grade (severe) [20,21]. 

### 2.4. Biochemical Evaluation 

Venous blood was collected after fasting overnight into vacutainer tubes (Becton & Dickinson, Plymouth, England) and centrifuged within 4 h. Serum glucose, total cholesterol, high density li-poprotein (HDL)-cholesterol, triglycerides, ALT, AST, GGT, total bilirubin, c-reactive protein (CRP) and insulin were measured with enzymatic colorimetric test [12]. Low-density lipoprotein (LDL) cholesterol level was calculated by the Friedewald formula [13]. Homeostatic model assessment (HOMA) index was calculated for assessing β-cell function and insulin resistance (IR) from fasting glucose and insulin concentrations [12]. Quality control was assessed daily for all determinations. 

### 2.5. Statistical Analysis

Data are expressed as mean ± standard deviation (SD). An unpaired Student’s *t*-test and Chi-squared test were used for comparisons of continuous and categorical variables, respectively. 

Change in CAP from baseline to follow-up (within group variation) was compared using paired Student’s *t*-test (two-tailed). An ANOVA test was used to compare the means between these three groups: (1) Improved (those who switched from a greater to a lesser steatosis grade); (2) Stable (those who remained in the same steatosis grade); and (3) Worsened (those who switched from a minor to a greater steatosis grade). 

To evaluate the ability of weight change (as %) to accurately identify worsened individuals over time, receiver–operating characteristic (ROC) curve analysis was performed.

Then, the outcome was measured with a univariate Kaplan–Meier model and overall strata comparisons measured by log–rank test. Multivariate Cox proportional hazard model was used to adjust for potential confounding factors, which were the variables correlated with CAP at Pearson’s correlation (age, BMI, glucose, liver stiffness) and medications. 

Significant differences were assumed to be present at *p* < 0.05 (two-tailed). With an expected effect size (ES) of 6.4 (mean difference of body weight between improved and worsened) and a standard deviation (SD) of 5, the standardized effect size (ES/SD) is greater than 1; for a two-tailed alpha equal to 0.05, the power of this study is 98%. 

All comparisons were performed using SPSS 22.0 for Windows (IBM Corporation, New York, NY, USA).

## 3. Results 

Table 1 shows the participants’ demographic and clinical characteristics. The mean age was 44 ± 13 years and time of follow-up 48 ± 3 months. The mean BMI was 25 ± 3 kg/m^2^ and a total of 49 individuals (55%) were overweight/obese at baseline. In this population, 66% had liver steatosis, 15% had severe steatosis (S3 grade) and 14% had type 2 diabetes mellitus (T2DM). Ulcerative colitis was present in 67% of the participants and Crohn’s disease in 33%. Only five participants (6%) had mild chronic active disease requiring a moderate-dose maintenance corticosteroid (CCS) for the relief of symptoms. Curative resection for Crohn’s disease was performed in four patients (all in the Stable group; 2 with Rutgeerts score of 1, and 2 with Rutgeerts score of 2, unchanged at follow-up). 

Table 2 shows the characteristics of the population at basal and follow-up according to the categorisation (improved, stable and worsened; see Supplementary Materials Figure S1). The three groups were comparable in term of age, gender, BMI and all other characteristics except for weight change (∆ = −1.0 ± 4; 2.5 ± 6 and 5.4 ± 5 kg, in improved, stable and worsened, respectively, *p* = 0.009 ), BMI change (improved vs. worsened, *p* = 0.002; post-hoc), CAP at follow-up (between groups, *p* < 0.001), basal liver stiffness (between groups, *p* = 0.021) and liver stiffness at follow-up (between groups, *p* = 0.015). 

We found that ROC area under the curve for the body weight change on predicting steatosis worsening was 0.68 (SE = 0.070; *p* = 0.017; confidence interval (CI) = 0.55–0.82) (figure not shown). A weight gain > 6% had a sensitivity of 53% and specificity of 81% in predicting steatosis worsening. Supplementary Materials Figure S2 shows the prevalence of the weight gain more than 6% in the three groups. 

**Figure 1:**
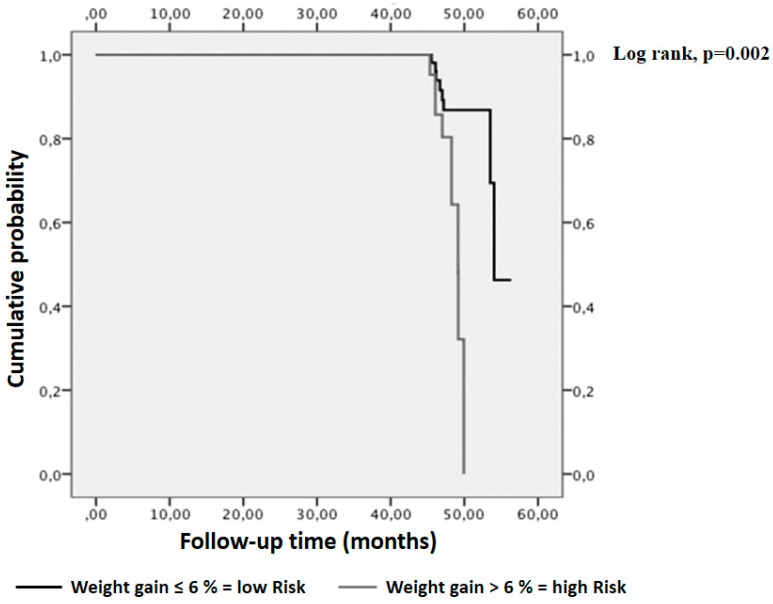
Impact of weight change on liver steatosis worsening in patients with IBD. Kaplan–Meier analyses demonstrated that a significantly higher liver steatosis deterioration event rate was observed with increasing body weight more than 6% (log–rank test, *p* = 0.002).

The Kaplan–Meier analysis demonstrated that a weight gain > 6% was associated with the greatest risk of worsening, over a period of four years, (log–rank (Mantel–Cox) χ^2^ test = 9.85; df = 1; *p* = 0.002, Figure 1).

In the Cox hazard analysis, only a weight gain > 6% increased the risk for worsening (HR 5.6; CI 1.46–22.11; *p* = 0.012) while age, BMI, medications, glucose and stiffness were not associated with the worsening of the liver steatosis (table not shown). 

## 4. Discussion

Limited research has been conducted studying the impact of dietary factors on liver steatosis in IBD patients. In this retrospective study, we observed a significant difference in weight change in a population of individuals affected by IBD who were categorised as improved, stable and worsened, by liver elastography, over a mean follow-up of four years. In particular, we found a greater probability of worsening in the hepatic fat content in individuals who gained more than 6% of body weight, over four years, than in those gaining less than this value. 

This is an unprecedented finding which, to the best of our knowledge, has never been investigated to date in patients with IBD and with a long-term follow-up. 

The pathogenesis of NAFLD is still incompletely understood. It is well recognized that weight-gain induces an accumulation of IHTG over time [3] and that obesity and weight gain are associated with liver disease progression [22]. It is well known that NAFLD is characterised by the accumulation of different lipid species, including toxic lipids, within hepatocytes [23] and it is now well accepted that the hepatocellular damage results from a complex balance involving liver, adipose tissue and gut [23]. In vitro and in vivo studies suggested that unutilized lipids lead to death of certain cells, processes referred to as “lipotoxicity” and “lipoapoptosis” that can reduce its life span [24,25,26]. During the development of diet-induced obesity, the adipocyte mass expands to store the surplus calories and leptin secretion rises acting on peripheral tissues by stimulating compensatory fatty acid oxidation [27]. This serves to partition the caloric surplus into white adipocytes. However, over time the leptin levels, although still elevated above normal, are not able to maintain sufficient compensatory oxidation in non-adipose tissues [27] with the generation of toxic lipid species that impact on the pathogenesis of liver steatosis [23]. It is now well accepted that weight loss and malnutrition are features of IBD patients during acute flare-ups and hospital admissions [28], while a normal or high BMI is associated with stable disease [29]. Over half of Crohn’s disease patients are overweight/obese [30]. Thus, there is now increasing focus on the potential negative effects of weight gain or obesity on the long-term general health of patients with IBD. In line with these studies, we found a prevalence of overweight/obese individuals of 55% and 65% at basal and follow-up. We assumed that patients in remission or with mild disease activity, who gained body weight, would be at high risk for liver steatosis, as in the general population. Our study suggests that a healthy diet and regular physical activity should be considered in individuals with IBD, which may help to prevent weight gain and certain serious complications. Furthermore, compared to IBD patients without liver disease, there is more than a twofold higher rate of inpatient morality among IBD patients with concomitant liver disease [31]. Thus, a healthy diet may improve survival among IBD patients. 

A previous study confirms our results since the authors evaluated the accumulation of fat in the liver, and the associated physiologic mechanisms, after a 6% weight gain over 12 weeks in a population of obese individuals [3]. However, the majority of studies have focused on the benefits of weight loss on hepatic steatosis and non-alcoholic steatotic hepatitis [32,33]. Our results are, thus, new since a weight reduction makes sense principally for overweight/obese individuals, while the strategies to prevent weight gain may be accessible and actionable for the majority of individuals, including those with a normal-weight. 

In this population of IBD patients, we found a liver steatosis prevalence of 66% [34]. This differs from a previous study in which the prevalence was 32.8% [10]. However, in the latter study the authors defined the presence of NAFLD with a CAP ≥ 248 dB/m, while we used a more inclusive cut-off [35]. 

In these individuals in clinical remission or affected by mild disease, we did not find an increased risk of liver steatosis with the use of medications such as, antiTNFα, mesalamine, azathioprine and corticosteroids but that only weight gain increased this risk. Despite the medications used in the treatment of IBD being involved in liver injury, several studies have demonstrated that the development of liver steatosis is independent of the medications [36] and that patients on solo immunosuppressive therapy with infliximab or solo therapy with azathioprine and with a longer IBD duration, had a higher risk of liver injury [37]. However, in this study, various strengths and weaknesses need to be addressed. First, the nature of our sample makes it difficult to generalize these results to all patients affected by IBD, but only to those with stable disease. Therefore, we are not able to completely exclude a deleterious effect of those drugs that are prescribed when patients are in active disease. Unfortunately, we did not have data on cytokines concentration. In addition, we have been able only to calculate the incidence of liver steatosis deterioration events at the end of the four years. It could be possible that a deterioration in liver steatosis occurred earlier in IBD patients who gained body weight, which was not captured in our study. Therefore, with this study we cannot provide information about the dynamics of the worsening of steatosis, that is, if it occurs early with respect to weight gain or late. Larger and more inclusive studies on this important issue are needed. Despite these limitations, our findings are original since to date, no studies exist assessing the effects of the weight change over time and liver fat content in IBD. The prospective design is a strength of this study as well as the availability of the measures of disease activity and data regarding the medications used. 

## 5. Conclusions

Evidence for weight gain to influence liver fat accumulation in individuals affected by IBD is relatively scarce. In this study, carried-out in patients with stable IBD and an overweight/obesity prevalence of 60%, we found that a gain of 6% in body weight over a period of four years increased the probability of having a deterioration in liver steatosis. In line with previous studies, we did not find an increased risk of liver steatosis with the use of medications. However, our results are applicable only to patients with stable disease. It could be speculated that changes in body weight will not completely mirror change in hepatic fat content due to the complex association between adiposity, medications and inflammation. Therefore, further studies are required to disentangle the individual components that influence the development of liver steatosis.

## Figures and Tables

**Table 1:** Baseline demographic and clinical characteristics of the population with Inflammatory Bowel Disease.

Variables	Mean ± SD
Age (years)	44 ± 13
IBD duration (years)	8 ± 7
Follow-up time (months)	48 ± 3
HBI	1.2 ± 1.9
MCI	0.9 ± 1.6
SES-CD	2.5 ± 1.2
Rutgeerts score *	1.5 ± 0.5
Weight (Kg)	71 ± 11
BMI (Kg/m^2^)	25 ± 3
Glucose (mg/dL)	95 ± 20
Insulin (μIU/ml)	11 ± 6
HOMA-IR	1.6 ± 1.7
Total Cholesterol (mg/dL)	180 ± 44
Triglycerides (mg/dL)	118 ± 70
HDL Cholesterol (mg/dL)	54 ± 17
LDL Cholesterol (mg/dL)	116 ± 33
AST (IU/L)	19 ± 7
ALT (IU/L)	21 ± 16
γGT (IU/L)	24 ± 17
Total Bilirubin (mg/dL)	0.63 ± 0.4
CAP score (dB/m)	237 ± 49
IQR (%)	11 ± 6
Liver Stiffness (kPa)	4.7 ± 1
IQR (%)	12 ± 7
*Prevalence*	
Gender (Male, %)	63
Smokers (%)	6
Crohn’s disease (%)	33
Ulcerative colitis (%)	67
Overweight/Obesity (%)	55
T2DM (%)	14
CCS (%)	6
antiTNFα (%)	18
5ASA (%)	93
AZA/6-MP (%)	18
Methotrexate (%)	1

Note: IBD = inflammatory bowel disease; HBI = Harvey Bradshaw index; MCI = Mayo clinic idex; BMI = body mass index; HOMA-IR = homeostatic model assessment for insulin resistance; HDL = high-density lipoprotein; LDL = low-density lipoprotein; AST = aspartate aminotransferase; ALT = alanine aminotransferase; γGT = gamma glutamyltransferase; CAP = controlled attenuation parameter; IQR = interquartile range; T2DM = type 2 diabetes mellitus; CCS = corticosteroids; antiTNFα = anti-tumour necrosis factor α antibodies; 5ASA = mesalamine; AZA/6-MP = azathioprine/6 -mercaptopurine. * four participants.

**Table 2:** Demographic and clinical characteristics of the Inflammatory Bowel Disease population according to the categorisation (improved, stable and worsened).

Variables	Improved ^a^ (*n* = 16)	Stable ^b^ (*n* = 56)	Worsened ^c^ (*n* = 17)	*p*-Value		Post-Hoc
Age (years)	42 ± 11	45 ± 13	45 ± 15	0.76		/
IBD duration (years)	9 ± 6	9 ± 7	7 ± 5	0.72		/
Follow-up time (months)	48 ± 4	47 ± 3	48 ± 3	0.46		/
HBI baseline	0 ± 0	1.6 ± 2	0.8 ± 1.8	0.21		/
HBI follow-up	0.60 ± 1.3	1.1 ± 1.9	2.0 ± 4.5	0.61		/
MCI baseline	1.3 ± 1.7	0.9 ± 1.8	0.7 ± 1	0.67		/
MCI follow-up	1.5 ± 2.4	0.7 ± 1.8	1.8 ± 2.2	0.18		/
SES-CD baseline	4 ± 1	2.2 ± 0.8	2.4 ± 1	0.017		a vs c 0.03 a vs b 0.005
SES-CD follow-up	4 ± 1	2.2 ± 0.8	2.4 ± 1	0.017		a vs c 0.03 a vs b 0.005
Weight baseline (Kg)	72 ± 8	71 ± 11	68 ± 10	0.52		/
Weight follow-up (Kg)	71 ± 10	73 ± 14	73 ± 10	0.72		/
Δ Weight (Kg)	−1.0 ± 4	2.5 ± 6	5.4 ± 5	0.009		a vs c 0.009
BMI baseline (Kg/m^2^)	26 ± 3	25 ± 3	24 ± 3	0.33		/
BMI follow-up (Kg/m^2^)	25 ± 3	26 ± 4	27 ± 4	0.46		/
Δ BMI (Kg/m^2^)	−0.8 ± 1	0.94 ± 3	2.5 ± 3	0.002		a vs c 0.002
Glucose (mg/dL)	98 ± 20	92 ± 18	104 ± 24	0.20		/
Insulin (μIU/ml)	13 ± 7	10 ± 6	10 ± 4	0.27		/
HOMA-IR	1.8 ± 1	1.6 ± 2	1.5 ± 2	0.94		/
Total Cholesterol (mg/dL)	180 ± 44	183 ± 44	167 ± 43	0.37		/
Triglycerides (mg/dL)	119 ± 50	123 ± 77	93 ± 50	0.27		/
HDL Cholesterol (mg/dL)	56 ± 15	54 ± 17	54 ± 17	0.93		/
LDL Cholesterol (mg/dL)	119 ± 40	116 ± 29	116 ± 43	0.92		/
AST (IU/L)	22 ± 6	19 ± 7	20 ± 7	0.28		/
ALT (IU/L)	23 ± 12	21 ± 18	17 ± 10	0.51		/
γGT (IU/L)	19 ± 7	26 ± 19	22 ± 19	0.30		/
Total Bilirubin (mg/dL)	0.63 ± 0.4	0.65 ± 0.4	0.57 ± 0.4	0.80		/
CRP (mg/dL)	3.6 ± 3	6.3 ± 14	3.9 ± 3	0.57		/
CAP score baseline (dB/m)	257 ± 34	236 ± 56	220 ± 24	0.092		/
CAP score follow-up (dB/m)	203 ± 33	234 ± 54	270 ± 21	<0.001		a vs c <0.001 b vs c 0.021
Δ CAP (dB/m)	−53 ± 21	−2.1 ± 18	51 ± 36	<0.001		a vs b <0.001 a vs c <0.001 b vs c <0.001
Liver Stiffness baseline (kPa)	4.1 ± 1	4.9 ± 1	4.7 ± 1	0.021		a vs b 0.021
Liver Stiffness follow-up (kPa)	4.2 ± 1	5.1 ± 1	5.5 ± 1	0.015		a vs c 0.018
Δ Stiffness (kPa)	0.15 ± 1	0.21 ± 1	0.76 ± 2	0.28		/
*Prevalence*						
Gender (Male, %)	75	63	53		0.42	
Smokers (%)	6	5	9		0.92	
Ulcerative colitis (%)	69	66	71		0.93	
Overweight/Obesity baseline (%)	69	55	41		0.28	
Overweight/Obesity follow-up (%)	50	66	77		0.27	
T2DM (%)	17	9	27		0.29	
CCS (%)	6	7	0		0.53	
antiTNFα (%)	19	20	12		0.75	
5ASA (%)	94	93	94		0.98	
AZA/6-MP (%)	31	14	18		0.29	
Methotrexate (%)	0	0	6		0.12	
Liver fibrosis (%)	0	5	12		0.36	

Note: IBD = inflammatory bowel disease; HBI = Harvey Bradshaw index; MCI = Mayo clinic index; Δ = change; BMI = body mass index; HOMA-IR = homeostatic model assessment for insulin resistance; HDL = high-density lipoprotein; LDL = low-density lipoprotein; AST = aspartate aminotransferase; ALT = alanine aminotransferase; γGT = gamma glutamyltransferase; CRP = C-reactive protein; CAP = controlled attenuation parameter; T2DM = type 2 diabetes mellitus; CCS = corticosteroids; anti TNFα = anti-tumour necrosis factor α antibodies; 5ASA = mesalamine; AZA/6-MP = azathioprine/6-mercaptopurine.

## Supplementary Materials

The following are available online at http://www.mdpi.com/2072-6643/11/2/303/s1, Figure S1: Prevalence in liver steatosis grade in patients with IBD, Figure S2: Prevalence of participants with a body weight gain > 6% according to the improved, stable and worsened liver fat content in IBD.
