# Supplementary material for: Weight Gain and Liver Steatosis in Patients with Inflammatory Bowel Diseases
Source: Nutrients. 2019 Feb 1;11(2):303. doi: 10.3390/nu11020303 (PMC6412993; doi:10.3390/nu11020303)
Supplement: Supplementary file 1 [file nutrients-11-00303-s001.pdf]

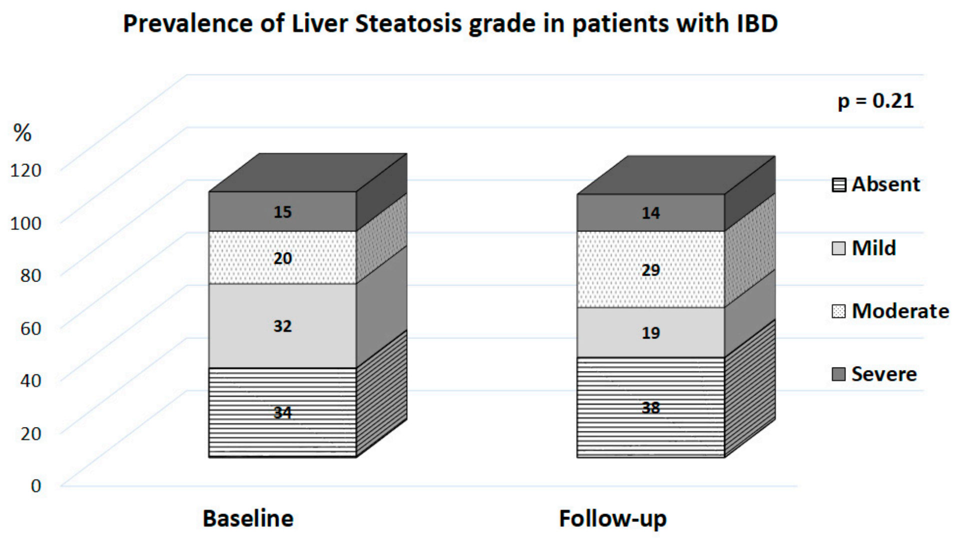

Figure S1. Prevalence in liver steatosis grade in patients with IBD

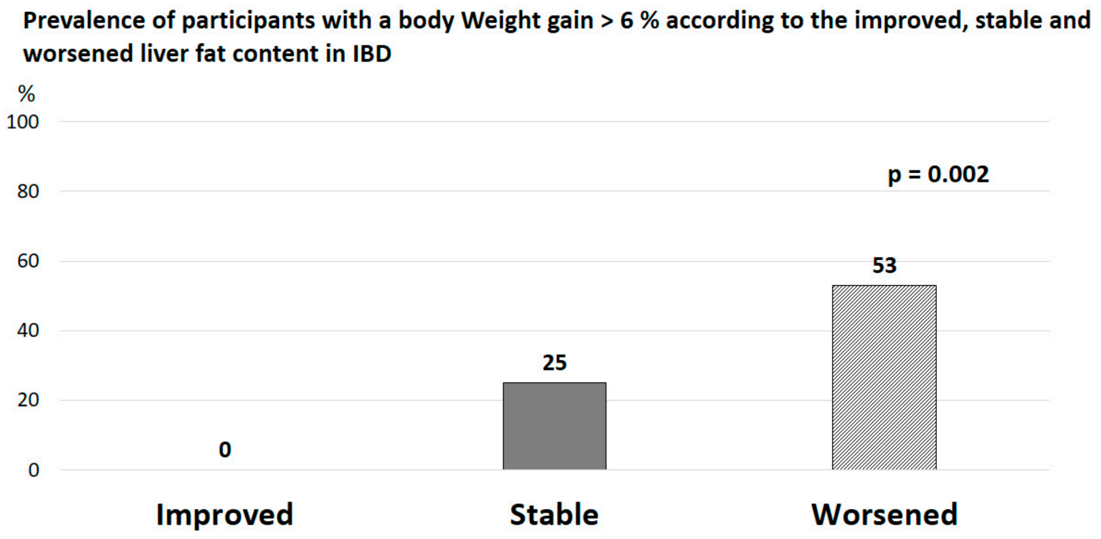

Figure S2- Prevalence of participants with a body weight gain > 6% according to the improved, stable and worsened liver fat content in IBD
